# MISC-1/OGC Links Mitochondrial Metabolism, Apoptosis and Insulin Secretion

**DOI:** 10.1371/journal.pone.0017827

**Published:** 2011-03-23

**Authors:** Marco Gallo, Donha Park, Dan S. Luciani, Katarzyna Kida, Ferdinando Palmieri, Oliver E. Blacque, James D. Johnson, Donald L. Riddle

**Affiliations:** 1 Department of Medical Genetics, The University of British Columbia, Vancouver, British Columbia, Canada; 2 Michael Smith Laboratories, The University of British Columbia, Vancouver, British Columbia, Canada; 3 Department of Cellular and Physiological Sciences, The University of British Columbia, Vancouver, British Columbia, Canada; 4 School of Biomolecular and Biomedical Science, UCD Conway Institute, University College Dublin, Belfield, Dublin, Ireland; 5 Department of Pharmaco-Biology, Laboratory of Biochemistry, University of Bari and Consiglio Nazionale delle Ricerche Unit for the Study of Mitochondria and Bioenergetics, Bari, Italy; Ecole Normale Supérieure de Lyon, France

## Abstract

We identified MISC-1 (Mitochondrial Solute Carrier) as the *C. elegans* orthologue of mammalian OGC (2-oxoglutarate carrier). OGC was originally identified for its ability to transfer α-ketoglutarate across the inner mitochondrial membrane. However, we found that MISC-1 and OGC are not solely involved in metabolic control. Our data show that these orthologous proteins participate in phylogenetically conserved cellular processes, like control of mitochondrial morphology and induction of apoptosis. We show that MISC-1/OGC is required for proper mitochondrial fusion and fission events in both *C. elegans* and human cells. Transmission electron microscopy reveals that loss of MISC-1 results in a decreased number of mitochondrial cristae, which have a blebbed appearance. Furthermore, our pull-down experiments show that MISC-1 and OGC interact with the anti-apoptotic proteins CED-9 and Bcl-x_L_, respectively, and with the pro-apoptotic protein ANT. Knock-down of *misc-1* in *C. elegans* and *OGC* in mouse cells induces apoptosis through the caspase cascade. Genetic analysis suggests that MISC-1 controls apoptosis through the physiological pathway mediated by the LIN-35/Rb-like protein. We provide genetic and molecular evidence that absence of MISC-1 increases insulin secretion and enhances germline stem cell proliferation in *C. elegans*. Our study suggests that the mitochondrial metabolic protein MISC-1/OGC integrates metabolic, apoptotic and insulin secretion functions. We propose a novel mechanism by which mitochondria integrate metabolic and cell survival signals. Our data suggest that MISC-1/OGC functions by sensing the metabolic status of mitochondria and directly activate the apoptotic program when required. Our results suggest that controlling MISC-1/OGC function allows regulation of mitochondrial morphology and cell survival decisions by the metabolic needs of the cell.

## Introduction

Mitochondria are the sites of enzymatic reactions fundamental for life, producing ∼95% of cellular ATP in eukaryotic cells. Mitochondria also play a dominant role in the control of apoptosis (reviewed in [1]). Several mitochondrial proteins are required for the intrinsic pathway of apoptosis. The succession of events from mitochondrial fragmentation to cell death has been well characterized and shown to be highly conserved from *C. elegans* to mammals [2], [3].

Mitochondria undergo constant rounds of fusion and fission to form or break their tubular structure within the cell. In addition to mitochondrial fragmentation during apoptosis [4], extensive mitochondrial remodelling happens in response to metabolic changes (reviewed in [5]). The exact mechanisms that control the interdependence of metabolic rate and mitochondrial structure are currently unknown. We investigated the possibility that mitochondrial metabolic proteins interact directly with members of the apoptotic machinery to control mitochondrial morphology and cell survival decisions. We focused on the mitochondrial 2-oxoglutarate carrier (OGC) to test our hypotheses.

The nuclear gene *OGC* encodes a monomeric carrier protein that resides in the inner mitochondrial membrane and is responsible for the electroneutral transport of α-ketoglutarate [6], [7]. This gene has been studied mostly for its effects on cellular metabolism. However, it has recently been shown that mammalian OGC imports about 40% of mitochondrial glutathione (GSH), a potent anti-oxidant peptide involved in oxidative stress response in mammals and *C. elegans*. Also, its over-expression in NRK-52E cells prevents chemically induced apoptosis [8]–[10]. These findings suggest that OGC might be involved in stress response as well as control of mitochondrial metabolism. In this report, we characterize the *C. elegans* homologue of *OGC*, which we called *misc-1* (Mitochondrial Solute Carrier), and find a novel role for MISC-1/OGC in apoptosis.

*C. elegans* has been used extensively to dissect the apoptotic pathway at the genetic level. Programmed cell death in *C. elegans* follows a linear genetic pathway involving *ced-9*/Bcl-2-like [11], *ced-4*/Apaf1 [12], [13] and *ced-3*/Caspase-9 [14]. Under non-apoptogenic conditions, CED-9 binds to CED-4 at the outer mitochondrial membrane to maintain it in its quiescent state [15]. Upon apoptotic stimuli, the BH3-only Bcl-2-family protein EGL-1 binds to CED-9 [16], which liberates CED-4 [17]. The now active CED-4 interacts with and activates the effector caspase CED-3 in the cytoplasm, thereby initiating a cascade of caspase activations that causes the hallmark phenotypes of apoptosis.

Mitochondria-induced apoptosis can result from the opening of channels called Mitochondrial Permeability Transition Pores (MPTPs), which are located at the junctions of inner and outer mitochondrial membranes. MPTP contributes to Mitochondrial Outer Membrane Permeabilization (MOMP) during apoptosis and allows exit of cytochrome c [18], [19] and other mitochondrial pro-apoptotic factors to the cytoplasm. MPTPs are multi-protein complexes, composed of the Voltage-Dependent Anion Channel (VDAC), the Adenine Nucleotide Translocase (ANT) and Cyclophilin D (Cyp-D; [20]–[22]). Multiple regulators have been identified, including the anti-apoptotic Bcl-2-family proteins Bcl-2 and Bcl-x_L_ [23], [18]. The identity of all MPTP components and regulators is not yet known. ANT is an established component of the MPTP in both mammals [24] and *C. elegans* [25]. We tested the possible interaction of OGC and MISC-1 with components of the MPTP.

Here we show that OGC and its *C. elegans* orthologue MISC-1 maintain the balance of fusion and fission events in human cells and in *C. elegans*. Genetically, the absence of MISC-1/OGC results in increased apoptosis rates through the physiological pathway. Furthermore, we provide genetic and molecular evidence of the involvement of MISC-1/OGC in control of insulin secretion and germline stem cell proliferation. Our work points to a novel, conserved and direct interaction between mitochondrial metabolic carriers and the apoptotic machinery to determine cell survival outcomes.

## Results

### *misc-1* expression

We investigated the expression profile of the 2-oxoglutarate carrier in *C. elegans*. Based on sequence similarity between MISC-1 and human OGC (72% amino acid identity; Supplementary Figure S1), their localization to mitochondria, and the shared roles of the two proteins (see below), we conclude that MISC-1 is the *C. elegans* orthologue of OGC.

**Figure 1:**
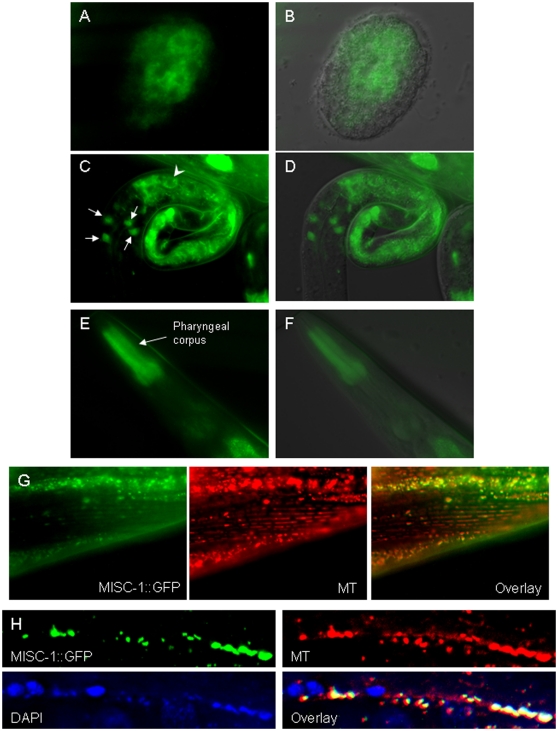
MISC-1::GFP reporter expression and its subcellular localization during *C. elegans* development. (**A–B**) GFP image and GFP/DIC overlay of MISC-1::GFP expression in an early embryo. Expression is in intestinal precursor cells and has a typically mitochondrial sub-cellular localization. (**C–D**) GFP image and GFP/DIC overlay of reporter expression in a newly hatched L1 animal. Expression can be observed in the intestine (arrowhead points to one typical cell) and in four head neurons (arrows). As in (A,B), the GFP reporter also has a string-like appearance typical of mitochondria. (**E–F**) GFP image and GFP/DIC overlay of reporter expression in an adult. Expression is strongest in the pharyngeal corpus. (**G**) Muscles of a MISC-1::GFP adult. MISC-1::GFP co-localizes with muscle mitochondria stained with the dye Mitotracker CMXRos (MT), as seen in the overlay. (**H**) Seam cell syncytium of a MISC-1::GFP adult. Mitochondria were stained with Mitotracker CMXRos and nuclei were counterstained with DAPI. The overlay shows the alternating pattern of mitochondria and nuclei in the seam cell syncytium, and illustrates how MISC-1::GFP co-localizes with the mitochondria.

We generated an extrachromosomal transgenic line carrying the full-length *misc-1* gene fused to *gfp* and under control of ∼1100 bp of the region 5′ of the ATG codon. Although low levels of reporter expression were observed ubiquitously (as expected), a subset of organs showed higher expression of MISC-1::GFP. GFP expression began early in embryogenesis in the intestinal precursor cells (Fig. 1A–B). The protein exhibited intestinal and neuronal localization in newly hatched L1s (Fig. 1C–D), while it was mainly present in the anterior pharynx in adults (Fig. 1E–F). A similar expression pattern was observed in a separate extrachromosomal transgenic line carrying a *misc-1* promoter GFP fusion construct (data not shown). Overall, this expression pattern suggests that MISC-1 is required in tissues with high energy demands, *i.e.* the intestine in developing animals and the pharynx in adults. This is consistent with the increased expression of our MISC-1::GFP reporter in calorie-restricted animals (Figure S2). It is interesting to note that in humans, *OGC* is most highly expressed in the heart (UCSC Genome Browser, [26]), another organ with high energy demands.

Although MISC-1/OGC is expected to be a mitochondrial carrier, we could find no published *in vivo* localization data for any organism. *C. elegans* embryonic and larval cells exhibited a typical mitochondrial pattern [27] for the localization of MISC-1::GFP (Fig. 1A–D). Co-localization of MISC-1::GFP and the mitochondria-specific marker MitoTracker Red CMXRos in muscle and seam cells indicates that MISC-1 is a mitochondrial protein (Fig. 1G–H).

### MISC-1 and OGC regulate mitochondrial morphology

**Figure 2:**
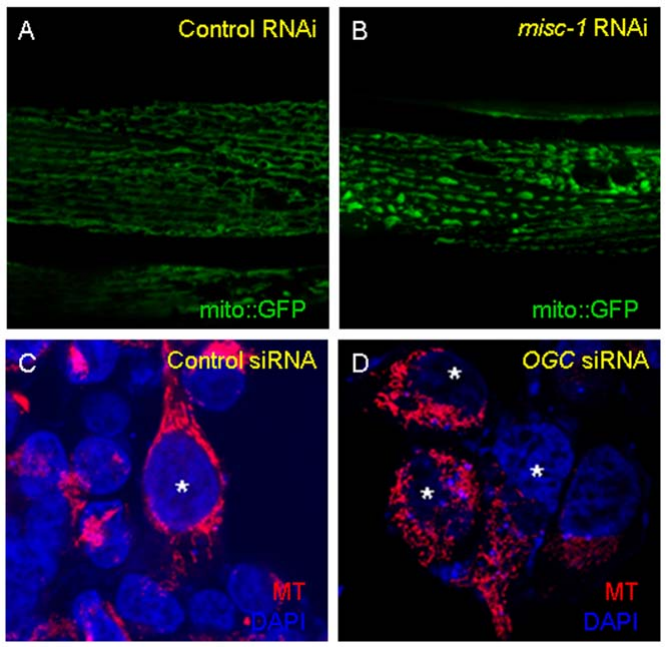
MISC-1/OGC is required for normal mitochondrial morphology in *C. elegans* and human cells. (**A**) Control RNAi and (**B**) *misc-1* RNAi on a transgenic strain carrying a muscle-specific, mitochondrially tagged form of GFP (mitoGFP). These confocal images show that *misc-1* knock-down causes mitochondrial fragmentation: the mitochondrial network breaks down because fission is more prevalent than fusion and total mitochondrial length is shorter than wild type. (**C**) Mitotracker staining and DAPI counterstaining of HEK293 cells transfected with the mammalian scrambled siRNA control vector. Mitochondria of transfected cells form organized networks, as expected. (**D**) Mitotracker staining and DAPI counterstaining of cells transfected with the *OGC* siRNA vector. These images show that downregulation of *OGC* in HEK293 cells results in mitochondrial fragmentation. *Transfected cells, as assessed by GFP fluorescence conferred by the transfected siRNA vector. MT: staining with MitoTracker CMXRos.

**Figure 3:**
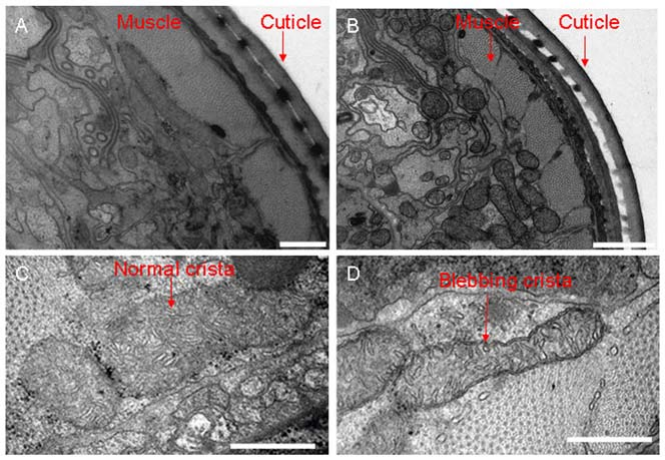
Mitochondrial ultrastructure is abnormal when MISC-1 is absent. Shown are low (**A–B**) and high (**C–D**) magnification images of TEM cross sections taken from the head region of N2 (A,C) and *misc-1* mutant (B,D) day 1 adults. Low magnification images show that compared with N2, the mitochondria of *misc-1* worms are smaller and more spherical. High magnification images indicate that compared to N2 controls, the mitochondrial cristae of the *misc-1* mutant are more disorganized, possess increased blebbing characteristics and adopt more irregular orientations. Furthermore, the cristae of *misc-1* animals are fewer in number. These images point to a function of MISC-1 in the stabilization of the inner mitochondrial membrane. The lower number of cristae in the absence of MISC-1 suggests that mutants have lower mitochondrial metabolic rates. Bars correspond to 500 nm in A, C and D, 1 µm in B.

Under normal cellular conditions, mitochondria are mostly found fused in branched structures. To assess the effects of *misc-1* knock-down on mitochondrial morphology, we used a transgenic line with GFP targeted to mitochondria and under control of the muscle-specific *myo-3* promoter (*myo-3*p::mito*::gfp*; [28]). *misc-1* RNAi resulted in robust (84%) down-regulation of MISC-1 protein levels (Figure S3) and was therefore deemed an appropriate system for the study of *misc-1* function. *misc-1* RNAi treatment resulted in mitochondrial fragmentation in the mito::*gfp* strain (Fig. 2A,B). In order to understand if this phenotype was specific to the mitochondria and not the result of *misc-1*-mediated defects in muscle fibres, we also tested the effect of *misc-1* RNAi on a strain carrying *myo-3*p::*myo-3*::*gfp* [29], a marker for the structure and organization of body wall muscle. Our results indicate that *misc-1* RNAi does not affect muscle structure (Figure S4; see also Fig. 3C–D). MISC-1 seems therefore to specifically control mitochondrial morphology.

To determine whether the effect of the 2-oxoglutarate carrier on mitochondrial morphology is conserved in mammals, we knocked down the *misc-1* human orthologue, *OGC*, in Human Embryonic Kidney 293 (HEK293) cells (Fig. S3). Staining with MitoTracker showed that siRNA against *OGC* resulted in a mitochondrial fragmentation phenotype reminiscent of *misc-1* knock-down worms (Fig. 2C,D). MISC-1 and OGC have the same effect on mitochondrial morphology in *C. elegans* and human cells, respectively.

Animals homozygous for the *misc-1(tm2793)* knock-out allele are morphologically normal, have the same developmental time as wild type (measured as time from egg laying to day one of adulthood), and have normal fat staining and lifespan (data not shown). However, when analyzed by transmission electron microscopy, we observed morphological defects in the mitochondria of *misc-1* animals. On average, the mitochondria of knock-out animals were reduced in size and more spherical in shape compared to N2 controls (Fig. 3A,B). Moreover, they displayed a reduced number of disorganized and abnormal mitochondrial cristae (Fig. 3C,D). These features suggest that absence of MISC-1 shifts the equilibrium of mitochondrial dynamics in the direction of fission and are in agreement with the mitochondrial fragmentation phenotype we observed with the mito::*gfp* strain. Furthermore, the absence of a key mitochondrial inner membrane protein disrupts normal formation or maintenance of cristae.

### Lack of MISC-1 results in increased germline apoptosis

**Figure 4:**
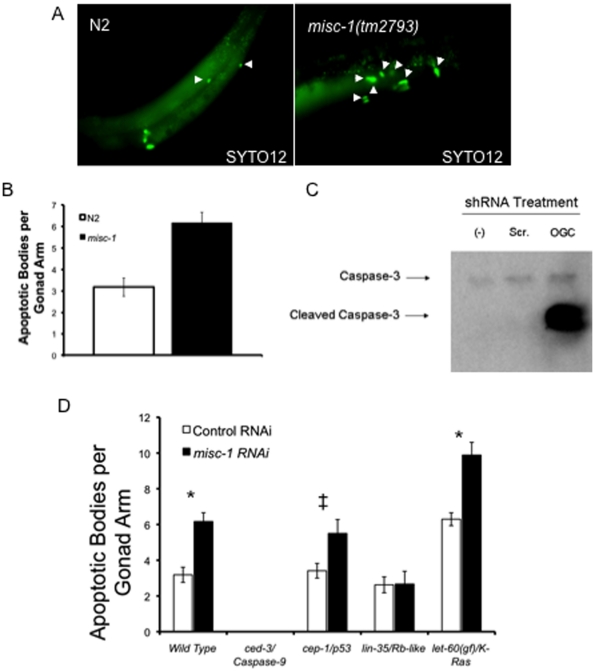
Absence of MISC-1 increases apoptosis in the *C. elegans* germline. (**A**) SYTO12 staining of apoptotic cell corpses (arrowheads) in the germline of N2 and *misc-1* knock-out. *misc-1* knock-out day 1 adults have an increased number of apoptotic events per gonadal arm. **(B**) Scoring of SYTO12-positive apoptotic corpses in N2 and *misc-1* knock-out. N2 had on average 3.2 apoptotic corpses per gonad arm, whereas *misc-1* had 6.2 per gonad arm. Absence of MISC-1 therefore results in a two-fold increase in apoptotic events in the germline. *t-test *P*<0.0001. N = 27 for N2 and N = 30 for *misc-1*. Error bars: ±SEM. (**C**) *OGC* knock-down induces apoptosis in MIN6 cells. MIN6 cells were either untransfected [(-) lane], transfected with a scrambled control shRNA vector (Scr. lane) or with an shRNA vector targeting mouse *OGC* (OGC lane). Cell lysates from the three treatments were used for a Western blot employing an antibody that recognizes both total Caspase-3 and cleaved Caspase-3. The latter is the active, pro-apoptotic form of the caspase and is therefore a marker of apoptosis. The Western blot shows that knock-down of *OGC* results in Caspase-3 activation and therefore apoptosis in MIN6 cells. Similar results were obtained with MIN6 cells at two different passages. (**D**) *misc-1* mediates apoptosis through the physiological pathway of apoptosis. Synchronized animals with different genetic backgrounds were fed either control or *misc-1* RNAi from the L1 stage and were stained with SYTO12 on day 1 of adulthood. SYTO12-positive apoptotic bodies per gonad arm were scored. *ced-3* mutants were used as a control, since mutations in this gene encoding an effector caspase obliterates germline apoptosis. *cep-1/p53* mutants suppress the DNA-damage pathway of apoptosis. *lin-35* mutants lack the physiological pathway of apoptosis. Worms carrying a *let-60/K-Ras* gain-of-function allele have increased apoptotic rates through the cytoplasmic stress pathway. The data indicate that mutations in *lin-35* suppress the apoptotic phenotype of *misc-1* RNAi, indicating that *misc-1* controls apoptosis through the physiological pathway of apoptosis. *t-test *P*<0.001. ^‡^t-test *P*<0.05.

Mitochondrial fragmentation is a hallmark of apoptosis in mammals and in *C. elegans* [4]. We used SYTO12 to stain apoptotic bodies in the adult germline in N2 and in the *misc-1* knock-out. We observed a two-fold increase in the number of apoptotic corpses per gonadal arm in *misc-1* knock-outs (Fig. 4A,B).

Since an increase in the number of apoptotic corpses could be caused either by increased apoptosis or by a defect in corpse removal, we used a transgenic line carrying a CED-1::GFP reporter [30] to monitor corpse engulfment. CED-1 is a receptor localized to the membrane of the sheath cells in the somatic gonad and is responsible for the recognition of cell corpses and the initiation of their engulfment and removal. We observed a two-fold increase in the number of corpses engulfed by CED-1::GFP(+) cells in animals treated with *misc-1* RNAi, compared to adults treated with control RNAi (Figure S5). These results indicate that *misc-1* RNAi does not impair the mechanism of cell corpse removal. We conclude that absence or decreased levels of MISC-1 increase the frequency of germline apoptosis.

In order to assess if reduced levels of OGC cause apoptosis in mammalian cells, we knocked-down mouse *OGC* in the MIN6 mouse insulinoma cell line [31] with an shRNA strategy. *OGC* knock-down caused robust cleavage and activation of Caspase-3, an established marker of activation of apoptosis (Fig. 4C). We conclude that lower levels of the 2-oxoglutarate carrier induce a phylogenetically conserved apoptosis program that is conserved from nematodes to mammals.

### *misc-1* acts through the physiological pathway of apoptosis

The increased levels of apoptosis observed upon knocking down *misc-1* were controlled at the genetic level, since a *ced-3(lf)*/Caspase-9 genetic background obliterated apoptosis caused by *misc-1* RNAi (Fig. 4D). In *C. elegans*, apoptosis can be triggered by three main pathways: (a) the DNA damage pathway, the main mediator being the *C. elegans* p53 orthologue CEP-1 [32]; (b) the cytoplasmic stress pathway, mediated by LET-60/K-Ras [33], and (c) the physiological pathway, mediated by the Retinoblastoma (Rb)-like protein LIN-35 [34]. All three pathways ultimately converge on the CED-9/CED-4/CED-3 core apoptotic machinery.

*misc-1* RNAi administered to wild-type N2 (Fig. 4D) recapitulated the germline apoptosis phenotype observed in *misc-1* knock-out worms (see Fig. 4B). This result, and the assessed efficiency of *misc-1* RNAi in down-regulating MISC-1 protein levels (Fig. S3), further confirms that RNAi is an appropriate tool to study *misc-1* function. We treated mutants in each of the three major apoptosis pathways with *misc-1* RNAi and assessed apoptosis levels by SYTO12 staining (Fig. 4D). The *cep-1(lf)/p53* background did not affect the ability of *misc-1* RNAi to increase the number of apoptotic corpses. Using the *let-60(n1046)/K-Ras* gain of function allele, which increases the basal rate of apoptosis through the cytoplasmic stress pathway, *misc-1* RNAi increased the apoptotic rate in an additive fashion (not synergistic), indicating that *misc-1* does not function through this pathway. By contrast, a *lin-35(lf)* mutant abrogated the apoptosis phenotype induced by *misc-1* RNAi. These results suggest that *misc-1* acts through the LIN-35/Rb-mediated physiological pathway of apoptosis.

### MISC-1 and OGC interact with proteins involved in the activation of apoptosis

**Figure 5:**
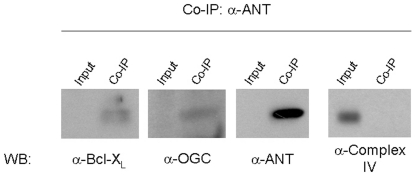
OGC interacts with Bcl-x_L_ and ANT. We performed a co-immunoprecipitation experiment in HEK293 cells using an antibody against ANT, which is an integral component of the MPTP. We were able to detect Bcl-X_L_, OGC and ANT in the co-immuno precipitant. As a control protein, we chose subunit 1 of complex IV of the electron transport chain, which is also localized to the inner mitochondrial membrane. This control protein did not co-immunoprecipitate with Bcl-x_L_, OGC and ANT, but was present in the supernatant (input). We obtained similar results by immunoprecipitation with an antibody against Bcl-x_L_ (data not shown). Co-IP: co-immunoprecipitation. WB: western blot.

Since we found that *misc-1* modulates mitochondrial fragmentation, cristae morphology and apoptosis, which are features also regulated by the MPTP, we asked if MISC-1 might interact with components or regulators of the MPTP, specifically Bcl-x_L_ and ANT. We performed a co-immunoprecipitation experiment using an antibody against ANT in HEK293 cells. ANT pulled down Bcl-x_L_ as expected, and it also precipitated OGC (Fig. 5). Likewise, Bcl-x_L_ co-precipitated ANT and OGC (data not shown). As a control, we tested a subunit of complex IV of the electron transport chain, which is also a mitochondrial inner membrane protein. This protein was not detected in the co-immunoprecipitated fraction; it was only present in the input/supernatant.

We observed similar interactions for MISC-1 in *C. elegans*. We did pull-down experiments in wild type and in our MISC-1::GFP over-expressing transgenic line with several combinations of antibodies and were able to confirm the interaction of MISC-1, CED-9/Bcl-2 and ANT (Figure S6). These pull-down experiments indicate that MISC-1 and OGC interact with proteins involved in the control of apoptosis.

### *misc-1* mutations suppress the dauer constitutive phenotype of *daf-2*

MISC-1 is a metabolic protein, controls mitochondrial function and interacts with CED-9, whose orthologue is Bcl-x_L_. Interestingly, transgenic mice with strong over-expression of Bcl-x_L_ in their pancreatic beta-cells were previously shown to have altered glucose-sensing at the mitochondria and impaired glucose-stimulated insulin release [35]. This observation suggests the possibility that Bcl-x_L_, like the related pro-apoptotic protein Bad [36], may play a role in insulin release independent of its role in apoptosis. We took advantage of the genetic tools afforded by *C. elegans* to investigate a possible role for *misc-1* in insulin signalling *in vivo*. The Insulin/Insulin-like Signalling (IIS) pathway is well conserved between *C. elegans* and mammals (reviewed in [37]). In *C. elegans*, the Insulin-like Growth Factor 1 Receptor (IGF1R) is encoded by *daf-2* (DAuer Formation) [38]–[40]. Mutations in this gene and reduced IIS result in dauer formation, which is a larval diapause state [41]–[43].

**Figure 6:**
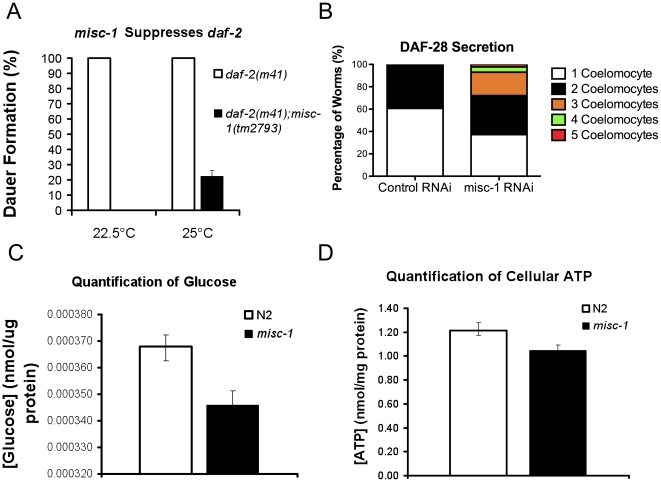
Absence of MISC-1 has positive effects on insulin secretion. (**A**) We assessed genetic interactions between *misc-1* and the hypomorphic allele *daf-2(m41)*. Mutations in *daf-2* result in constitutive dauer formation at 22.5°C and 25°C. However, the double mutant *daf-2;misc-1* worms did not arrest at the dauer stage at 22.5°C, and only 22.5% of the double mutants formed dauer larvae at 25°C. These data show that absence of *misc-1* expression suppresses the dauer phenotype conferred by *daf-2* mutations. T-test *P* = 0.0021. (**B**) We tested the possibility that the *daf-2* suppression by *misc-1* was due to an increase in insulin secretion, which could over-ride the hypomorphic allele *m41*. We measured insulin secretion by scoring GFP(+) (*i.e.* DAF-28::GFP-positive) coelomocytes in a transgenic strain upon *misc-1* knock-down. *misc-1* RNAi animals (N = 44) had on average more GFP(+) coelomocytes than control worms (N = 33) (two-way ANOVA *P* = 0.04). These data suggest that *misc-1* knock-down increases insulin secretion. (**C**) We quantified glucose concentration in N2 wild type or *misc-1* knock-out animals, and observed a 6% reduction in glucose concentration in *misc-1* mutants, compared to control (t-test *P* = 0.0054). The amount of glucose was normalized to protein content. (**D**) We quantified the amount of cellular ATP in N2 and *misc-1* mutants, and observed no significant difference in the concentration of ATP between the two strains (t-test *P* = 0.09).

To test the possibility that MISC-1 might play a role in insulin secretion in *C. elegans*, we first tested a possible genetic interaction between *misc-1* and *daf-2/IGF1R*. We created a double mutant carrying the hypomorphic allele *daf-2(m41)* and the knock-out allele *misc-1(tm2793)* to ask whether *misc-1* modified the *daf-2* dauer-constitutive phenotype (Fig. 6A). *m41* animals constitutively enter the dauer diapause stage at non-permissive temperatures (>22°C). All *daf-2* animals entered the dauer stage at both 22.5°C and 25°C, but no *daf-2;misc-1* animals entered the dauer stage at 22.5°C and only 22% formed dauer larvae at 25°C. Therefore *misc-1* substantially suppresses the dauer phenotype of *daf-2*. The suppressive effects of the mutation are stronger at lower temperatures, probably because *m41* is a temperature-sensitive allele with a stronger phenotype at higher temperatures.

### MISC-1 modulates DAF-28 secretion

The suppression of the *daf-2* dauer-constitutive phenotype by *misc-1(tm2793)* could be interpreted in at least two ways: (a) *misc-1* could be genetically downstream of *daf-2* or part of a pathway downstream of the insulin-like pathway; (b) mutations in *misc-1* may positively affect insulin secretion, which in turn would compensate for the partial loss of DAF-2 function due to the hypomorphic mutation *m41*. To address this issue, we employed a strain carrying a translational fusion of the *C. elegans* insulin DAF-28 and GFP (DAF-28::GFP; [44]). We subjected the DAF-28::GFP strain to *misc-1* RNAi by feeding. DAF-28::GFP is secreted by the head neurons and the posterior intestine into the pseudo-coelomic fluid, where it is picked up by scavenging cells called coelomocytes. Among a total of six coelomocytes, the number containing DAF-28::GFP (GFP(+)) is proportional to the amount of secreted insulin [44]. When we compared the number of GFP(+) coelomocytes in worms treated with control and *misc-1* RNAi, we observed that knock-down of *misc-1* induced an increase in GFP(+) coelomocytes (average of two independent experiments; Fig. 6B). In control RNAi animals, we observed no more than two GFP(+) coelomocytes, whereas we observed three GFP(+) coelomocytes in 21% of *misc-1* RNAi-treated adults, four GFP(+) coelomocytes in 4.6% of the adultss and five GFP(+) coelomocytes in 2.3% of the adults observed. The results show a statistically significant increase in DAF-28::GFP secretion in worms treated with *misc-1* RNAi (*P*<0.05 by two-way ANOVA).

The DAF-28 protein acts as a DAF-2 receptor agonist, but the *C. elegans* genome encodes over 40 insulin-like proteins, some being agonists and some antagonists of DAF-2 [44]–[46]. To corroborate the DAF-28::GFP reporter results as a general quantitative marker of insulin secretion, we measured the amount of glucose in *misc-1* and wild type N2 mixed-stage populations. We reasoned that if indeed insulin secretion is increased in *misc-1* mutants, then their glucose utilization should increase and the total glucose level should drop, compared to normal animals. In fact, we observed a 6% decrease (t-test *P* = 0.0054) in glucose levels in the *misc-1* mutant (Fig. 6C), a result consistent with an increase in insulin secretion when MISC-1 levels are reduced. Organismal levels of ATP are not affected by absence of MISC-1 (Fig. 6D), indicating that ATP may not be responsible for the induction of insulin secretion in the case of *misc-1* insufficiency.

### *misc-1* activity negatively modulates germline proliferation

The *C. elegans* germline is a syncytium of nuclei that mature as they migrate from a distal to a proximal position and eventually become oocytes. Notch signaling initiated from the Distal Tip Cell (DTC) regulates the balance between proliferation (mitosis) and differentiation (meiosis) of these germline nuclei. The DTC produces the Notch ligand LAG-2 [47]–[49], which activates the GLP-1/Notch receptor in nearby germ cells and maintains these cells in the undifferentiated (mitotic) state [50], [51]. A recent report showed that increased insulin signalling can increase germ cell proliferation independently of LAG-2 activation [52]. A similar effect of insulin signalling on female reproduction was observed in mouse [53].

**Figure 7:**
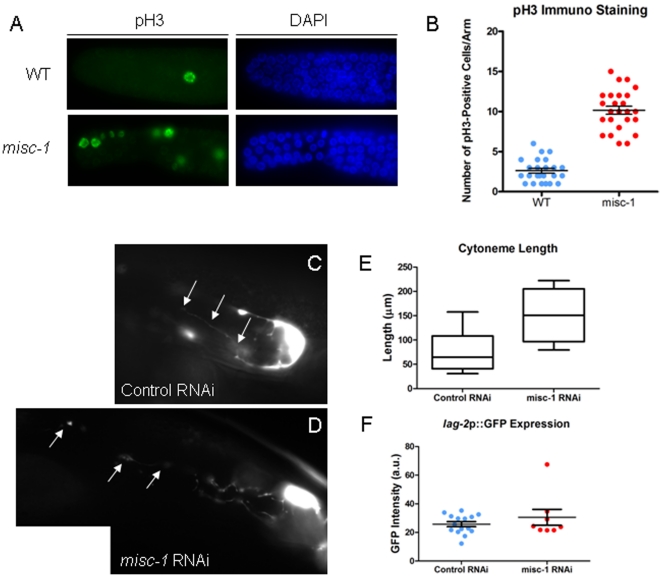
Absence of MISC-1 positively affects germline proliferation. (**A**) Immunostaining with α-phospho-H3 (pH3, left panel) and DAPI staining (right panel) of dissected germlines. Nuclei positive for phospho-H3 are actively undergoing mitosis. We observed an increase in mitotic cells in the germline of *misc-1* knock-out animals, compared to wild type (WT) control. (**B**) Quantification of phospho-H3-positive nuclei in dissected germlines of wild type (WT) and *misc-1* knock-out animals. While wild type had on average 2.63 pH3-positive nuclei, *misc-1* worms had 10.2 pH3 positive cells per gonadal arm (t-test *P*<0.0001). (**C–D**) Transgenic animals expressing a *lag-2*p*::gfp* reporter were exposed to either control (empty) RNAi vector (C) or *misc-1* RNAi (D). *misc-1* knock-down results in longer DTC cytonemes than control (arrows delineate the length of the cytonemes). (**E**) Measurement of cytoneme length in control animals and animals exposed to *misc-1* RNAi. The mean cytoneme length was 74.9 µm in control, while it was 150.9 µm in *misc-1* knock-down worms. Cytonemes in *misc-1* knock-down worms were therefore twice as long as in control (unpaired t-test *P* = 0.0080). (**F**) Absence of MISC-1 does not affect *lag-2* expression. We quantified reporter expression with ImageJ. We observed no difference in GFP expression between control worms and *misc-1* RNAi-treated worms (t-test *P* = 0.3137). All error bars represent ±SEM.

We investigated the possibility that the increased insulin secretion in *misc-1* mutants could have an effect on germline proliferation. Proliferative germ cells in late prophase and early mitotic M-phase are positive for histone 3 (H3) phosphorylation (pH3; [54], [55]). We performed immunostaining of dissected germlines from wild type and *misc-1* knock-out mutants with a pH3 antibody. The results show that absence of MISC-1 greatly increases the number of pH3-positive, mitotic germline stem cells (Fig. 7A,B) and causes an extended mitotic zone (data not shown). pH3 immunostaining strongly suggests that absence of MISC-1 favours mitosis (self-renewal) over meiosis (differentiation) in germline stem cells.

To assess the involvement of LAG-2, we employed a transgenic line expressing a GFP reporter under the control of the *lag-2* promoter (*lag-2*p::*gfp*; [56]). This reporter is expressed in the DTCs and its processes, called cytonemes (Fig. 7C,D). We found that the cytonemes of animals treated with *misc-1* RNAi were longer than the ones in control animals (Fig. 7C–E). Knock-down of *misc-1* resulted in a two-fold increase in the length of the cytonemes (mean ± SEM: control RNAi = 74.9±10.80 µm, *misc-1* RNAi = 150.9±29.14 µm; t-test *P* = 0.0080). We assessed *lag-2*p::*gfp* expression by microscopy in animals treated with control or *misc-1* RNAi, but were unable to detect statistically significant differences between the two treatments (Fig. 7F). We concluded that upregulation of *lag-2* was not responsible for the extended cytonemes and mitotic zones of *misc-1* mutants. Although the length of the cytonemes may be positively correlated with the number of germline cells in a mitotic state, this is independent of *lag-2* expression. The germline stem cell proliferation phenotype obtained by lack of MISC-1 is therefore consistent with an increase in insulin signalling.

## Discussion

We identified *misc-1*, the *C. elegans* orthologue of human *OGC*. By studying *misc-1* in nematodes, we uncovered new functions for this gene that are conserved in higher eukaryotes. We showed that MISC-1/OGC is not merely involved in regulation of mitochondrial metabolism by participating in the malate-aspartate shunt. The protein seems to participate in a higher order of metabolic regulation by interacting with Bcl-2-family members and by regulating insulin secretion. Specifically, our experiments in *C. elegans* and mammalian cells show that MISC-1 and OGC regulate mitochondrial fusion/fission, cell death and interact with regulators of apoptosis.

First, we showed that reducing or eliminating expression of MISC-1/OGC resulted in mitochondrial fragmentation in *C. elegans* and in human cells. The mitochondrial fragmentation phenotype and the concomitant reduction in cristae number in *misc-1* knock-out animals suggest a reduction in mitochondrial metabolism (reviewed in [5]). Mitochondrial metabolism also decreases and cristae condense in the absence of the fusion machinery [57]. For instance, loss of function of Mitofusin-2 (*Mfn2*) – a key protein involved in mitochondrial fusion - in cell cultures inhibits pyruvate, glucose and fatty acid oxidation and reduces mitochondrial potential [58]. *Mfn2* expression is down-regulated in the Zucker rat model of obesity [57], [58] and in human subjects with obesity or diabetes [59]. Hence, mutations affecting mitochondrial structure also affect metabolism. Here we provide an example that the opposite is also true: a metabolic protein affects mitochondrial structure.

The mitochondrial fragmentation conferred by *misc-1* knock-out or knock-down could be mediated by its physical interaction with CED-9/Bcl-2, which is a component of the core apoptotic machinery. The fact that knocking-down *misc-1* in *C. elegans* and *OGC* in human cells resulted in the same phenotype – mitochondrial fragmentation – suggests that MISC-1/OGC might be part of an ancient, phylogenetically conserved mechanism to induce mitochondrial fragmentation. This hypothesis is strengthened by the observation that CED-9 physically interacts with FZO-1/Mfn1,2 to promote mitochondrial fusion [60].

The interaction of MISC-1/OGC with CED-9/Bcl-2 may not be unique, as other mitochondrial proteins localized to the inner mitochondrial membrane might similarly interact with Bcl-2-family members in order to regulate the mitochondrial fusion/fission equilibrium according to metabolic needs. An example of such a protein is ANT, the primary role of which is to mediate the exchange of ADP and ATP across the inner mitochondrial membrane. MISC-1/OGC and ANT are of similar size, share protein domains (three solute carrier or SolCar domains), and are both localized to the inner mitochondrial membrane [61].

Our data suggest that MISC-1 and OGC participate in an evolutionarily conserved apoptosis program. Furthermore, we showed that *misc-1* modulates apoptosis through the physiological (LIN-35/Rb) pathway. This pathway is not activated by stress, but functions in the *C. elegans* germline during oocyte production [33], [34], similarly to apoptosis during oocyte production in humans. It has been shown that the physiological pathway activates the core apoptotic machinery through an unknown mechanism, independent of the BH3-only protein EGL-1 [33]. We hypothesize that the physiological pathway of apoptosis is triggered by the efflux of mitochondrial pro-apoptotic factors controlled by the function of MISC-1/OGC and the MPTP.

It has been suggested that the MPTP might function as a low-affinity Ca^2+^ channel [62]. It is interesting to speculate that Ca^2+^ may mediate the apoptosis and insulin secretion phenotypes observed in the absence MISC-1/OGC, since we were unable to detect an increase in ATP levels in whole-organism extracts from *misc-1* mutants that exhibited higher insulin secretion. It would be interesting to measure ATP levels specifically in the *C. elegans* insulin-secreting cells. However, because of the lack of techniques to dissect single tissues in the adult, we measured total ATP levels, thereby possibly diluting any effect *misc-1* mutations might have on ATP production in insulin-producing cells. Whether through release of mitochondrial Ca^2+^ or not, we offer genetic and molecular evidence that absence of MISC-1 results in increased insulin secretion in *C. elegans*.

Our experiments do not distinguish whether MISC-1 has effects on secretion of only DAF-28 or if it is a more general modulator of insulin secretion. Further studies should be undertaken to clarify this issue. α-ketoglutarate, the main substrate of MISC-1/OGC, is an insulin secretagogue (reviewed in [62]). Furthermore, over-expression of Bcl-x_L_ in mouse β-cells impairs glucose- and KCl-stimulated insulin secretion and intracellular Ca^2+^ levels [35]. Bcl-2-proteins could modulate insulin secretion via release of mitochondrial and ER Ca^2+^ [63]. It is therefore possible that MISC-1/OGC affects insulin secretion by modulating the function of Bcl-2 proteins, probably through regulation of Ca^2+^ release from mitochondrial stores. Interestingly, an expression microarray study [64] showed that the gene encoding OGC is also down-regulated in muscles of individuals affected by type 2 diabetes or with a family history of the disease, compared to controls.

Absence of MISC-1 favours mitotic proliferation of germline stem cells. This result seems to further corroborate the increase in insulin secretion in *misc-1* knock-down and knock-out animals. In fact, increased insulin secretion and insulin signalling induces germline proliferation in *C. elegans*, independently of the Notch ligand LAG-2 [52]. Here we show that absence of MISC-1 induces germline stem cell proliferation independently of LAG-2. We considered the possibility that increased germline stem cell proliferation may represent a compensatory mechanism for the increased apoptotic rate in the germline of *misc-1* animals. However, several reports suggest that germline stem cell proliferation and apoptotic rates are not interdependent. For example, absence of the germline RNA helicase CGH-1 results in increased apoptosis and decreased germline proliferation [65], whereas absence of the RNA binding protein GLD-1 results in reduced apoptosis and increased germline proliferation [66]. We speculate that increased insulin secretion stimulates the growth of DTC cytonemes and that longer cytonemes are responsible for maintaining the extended mitotic zone observed in *misc-1* germlines. To the best of our knowledge, such an association between cytoneme length and germline stem cell proliferation has not been reported before.

In conclusion, we identified MISC-1/OGC as a novel regulator of mitochondrial fusion/fission, apoptosis, insulin secretion and germline proliferation. The functions of this protein seem to be phylogenetically conserved from nematodes to mammals. Our data suggest that MISC-1/OGC has a role in assessing the metabolic status of a cell and subsequently in making cell survival decisions. We find it intriguing that a primarily metabolic protein like MISC-1/OGC plays roles in such a wide array of functions. The study of other metabolic mitochondrial carriers in the context of their wider cellular functions might elucidate the complex mechanisms underlying the integration of cell survival signals with cell metabolism.

## Materials and Methods

### Strains

The following strains were used: N2, *daf-2(m41)* (DR41), *misc-1(tm2793)* (DR2520), *daf-2(m41);misc-1(tm2793)* (DR2540), *daf-28*p::*daf-28::gfp*, *ced-1::gfp* (MD701), *ced-3(n717)*, *cep-1(gk139)*, *lin-35(n745)*, *let-60(n1046)*, *mgIs[rol-6(su1006);myo-3*::mito::*gfp*] (DM8006), *myo-3*p::*myo-3*::*gfp* (RW1596), *qIs56[lag-2p::gfp; unc-119(+)]*. All strains were maintained on agar plates spread with *E. coli OP50* using standard techniques [67]. DR2520 was obtained by out-crossing the original knock-out strain received from the Knock-out Consortium three times with wild-type N2.

### Cell culture

HEK293 and MIN6 cells were cultured in Dulbecco/Vogt modified Eagle's Minimal Essential Medium (DMEM) containing 25 mM glucose, 10% heat-inactivated Fetal Calf Serum (FCS) and 1% penicillin/streptomycin at 37°C and 5% CO_2_.

### Transgenic lines

For the transgenic line carrying the MISC-1::GFP reporter, we amplified 1165 bp upstream of the *misc-1* ATG codon and the full *misc-1* gene from N2 genomic DNA. We then fused *misc-1* to *gfp* using the stitching PCR method [68]. The transgenes were injected with the *rol-6(su1006)* co-injection marker in the gonads of N2 hermaphrodites according to established protocols [69]. Although the transgene was not integrated in the genome, ∼90% of animals in each generation carried the transgene.

### RNAi, siRNA and shRNA

RNAi by feeding was performed as previously described [70], [71]. The clone used for *misc-1* RNAi was B0432.4. *misc-1* RNAi and the empty RNAi vector (L4440; [70]) were propagated in *Escherichia coli* HT115 and administered to worms according to established feeding RNAi protocols [72]. For siRNA in HEK293 cells, we designed oligos targeting *OGC* with the software OligoEngine Workstation. The oligos were annealed in the siRNA vector pSuper.gfp/neo (OligoEngine) to make pSuperOGC. 1 µg of pSuperOGC or a control vector containing a scrambled mammalian sequence (X-Scramble) were transfected in HEK293 cells using Lipofectamine (Invitrogen). HEK293 transfection with Lipofectamine was done for 6–8 h at 37°C. Cells were grown for 24–48 h before being used for microscopy or RNA extraction. For *OGC* knock-down in MIN6 β-cells, an shRNA vector targeting mouse *OGC* was employed (TG504014, OriGene). The cells were grown in 24 well plates and transfected for 6–8 hours using 500 ng of either *OGC* shRNA vector or a vector containing a scrambled shRNA cassette as control and 1 µl Lipofectamine 2000 (Invitrogen) in 500 µL Opti-MEM, according to the manufacturer's instructions. Following transfection, MIN6 cells were cultured in DMEM with no penicillin/streptomycin for 72 hours before the assessment of apoptosis activation.

### Microscopy

Worms were mounted on a 3% agarose pad and immobilized with 10 mM Levamisol (Sigma-Aldrich). Fixed HEK293 cells were mounted on microscope slides using the Vectashield mounting solution (Vector Laboratories). Images were taken using a Zeiss Axioskop with a QImaging Retiga 2000R camera. The imaging software used was Openlab 5.5.0. For some images (Fig. 1G–H, Fig. 2), we used a Zeiss Axiovert 200 M confocal microscope with the LSM 5 Pascal laser system. Quantification of GFP reporter expression was performed with ImageJ (Rasband WS, ImageJ, U. S. National Institutes of Health, Bethesda, Maryland, USA, http://rsb.info.nih.gov/ij/, 1997–2009.).

### Electron microscopy

Adult worms were washed directly into a primary fixative of 2.5% glutaraldehyde in 0.1 M Sorensen phosphate buffer. Worms were transferred to microcentrifuge tubes and fixed for one hour at room temperature. Samples were then centrifuged at 3,000 rpm for one minute, supernatant removed and washed for ten minutes in 0.1 M Sorensen phosphate buffer. The worms were then post-fixed in 1% osmium tetroxide in dH2O for one hour at room temperature. Following washing in buffer, specimens were processed for electron microscopy by standard methods; briefly, they were dehydrated in ascending grades of alcohol to 100%, infiltrated with Epon and placed in aluminum planchetes orientated in a longitudinal aspect and polymerized at 60°C for 24 hours. Using a Leica UC6 ultramicrotome, individual worms were sectioned in cross section at 1 mm intervals, starting outside the worm, until the anterior tip of the animal was located as judged by examining the sections stained with toluidine blue by light microscopy. Thereafter, serial ultra-thin sections of 90 nm were taken. Sections were picked onto 200 mesh copper grids, stained with uranyl acetate and lead citrate and examined under a Tecnai Twin (FEI) electron microscope. All mitochondrial images were taken from muscle cells observable in anterior sections of the worm (*i.e.*, typically 5–15 mm from the nose tip). Where required, sections were tilted using the Compustage of the Tecnai to ensure an exact geometrical normalcy to the imaging system. All images were recorded at an accelerating voltage (120 kV) and objective aperture of 10 µm, using a MegaView 3 digital recording system.

### qRT-PCR

L1 larvae synchronized by sodium hypochlorite treatment and overnight starvation in M9 buffer were spotted on plates and grown until day 1 of adulthood. RNA was extracted with TRIzol (Invitrogen) and chloroform and purified with RNeasy Mini Kit (Quiagen). Equimolar concentrations of control and experimental RNA were used to make cDNA with SuperScript II Reverse Transcriptase (Invitrogen). Amplification of target genes was done with iTaq SYBR Green Supermix with ROX (Bio-Rad) using an AB 7500 Fast Real-Time PCR System with standard settings. Target gene expression was normalized against endogenous levels of *act-2* expression. The same protocol was followed for qRT-PCR experiments with HEK293 cells and normalization was done with respect to *Actβ* expression.

### Co-immunoprecipitation and Western blots

HEK293 cells were harvested in co-immunoprecipitation buffer (25 mM TRIS pH 7.4, 100 mM NaCl, 1 mM EDTA, 0.5% NP-40, 1% sodium deoxycholate) with protease inhibitor cocktail (#539134, Calbiochem). Following sonication, the solution was pre-cleared with protein G-sepharose at slow rotation for 1.5 h at 4°C. The antibodies targeting the protein to be co-immunoprecipitated were then added at a concentration of 1∶50 and allowed to bind at slow rotation at 4°C overnight. The next morning protein G-sepharose beads were added and the immunoprecipitation was allowed to run at slow rotation for 4 h at 4°C. Beads were washed four times, Laemmli buffer was added to the beads and the protein samples were heated at 65°C for 10 min before being loaded on an SDS-PAGE gel. Western blots were performed using standard techniques.

We used a similar protocol for *C. elegans* co-immunoprecipitation. Briefly, mixed stage worms were frozen at −80°C in co-immunoprecipitation buffer. The samples were then thawed on ice and sonified four to five times (Sonifier 450 settings: output 3 for 10 s). The lysates were then used for co-immunoprecipitation as described above for mammalian cells. For Western blots using *C. elegans*, we boiled the samples for 10 min in Laemmli buffer. We generated an α-MISC-1 antibody with GenScript Corporation. Other antibodies used: OGC (developed by FP); Bcl-xL (#2762, Cell Signaling Technology); α-ANT (# MSA02) and α-Complex IV subunit 1 (# MS404, MitoSciences). Western blots for activation of apoptosis employed a Caspase-3 antibody (#9662, Cell Signaling).

### Staining *C. elegans* and HEK293 mitochondria

Mitochondria were stained with MitoTracker CMXRos (Invitrogen). To stain mitochondria in worms, we resuspended MitoTracker in molten (55°C) NG agar to a final concentration of 2 µg/mL. We poured 3–4 mL of the solution in 50 mm plates. The plates were then spotted with 100 µL of OP50. Plates were stored in the dark before and during the experiment. Synchronized L1 larvae were spotted on the plates and allowed to grow until used for microscopy on day 1 of adulthood. To stain mitochondria in HEK293 cells, we added MitoTracker CMXRos to fresh growth medium at a final concentration of 100 nM. The cells were incubated at 37°C for 45 min, fixed and counterstained with DAPI (4′,6-diamidino-2-phenylindole, Sigma) according to suppliers' protocols.

### Germline apoptosis and DAPI staining and phospho-H3 immunostaining

To stain apoptotic bodies in the germline, we used the dye SYTO12 green fluorescent nucleic acid stain (Invitrogen) according to established protocols [33].

DAPI staining was done following standard techniques. Phospho-H3 immunostaining was performed as previously described [73].

### Metabolic assays

Glucose concentration in worms was measured with a Glucose Assay Kit (#K606-100, BioVision) according to manufacturer's instructions. Briefly, synchronized day-one adults were washed 5 times in M9 buffer. The worm pellet was resuspended in 500 mL of the glucose assay buffer provided in the kit, frozen for storage at −80°C, thawed on ice and sonified (Sonifier 450) five times (output level 3, time 10 s) to obtain extracts. After centrifugation at 13,000 rpm at 4°C, the supernatant was used for the glucose assay as per manufacturer's manual. The glucose concentration was normalized to protein content in each sample. The lysates for ATP analysis were generated from synchronized day-one adults, which were frozen, sonicated as described above, and boiled for 15 min. ATP contents were assayed with the Adenosine 5′-triphosphate (ATP) Bioluminescent Assay Kit (#FL-AA, Sigma) according to manufacturer's instructions. Measurements of signal intensity were taken with a Tecan M200 micro plate reader.

### Statistical analyses

Results are expressed as mean ± S.E.M. A *P*-value of less than 0.05 was considered significant. Statistical analyses were performed with the statistical package GraphPad Prism 5.02.

## Supporting Information

Figure S1 **Amino acid conservation between** ***C. elegans*** **MISC-1 and human OGC.** MISC-1 and OGC share 72% identity and 80% similarity (e-value = 8e^−105^) at the amino acid level. The two proteins are best reciprocal matches. When doing BLAST similarity searches with the MISC-1 sequence, the second best human match is the dicarboxylate carrier, with an expect value of 2e^−51^, amino acid identity of 39% and similarity of 57%. BLAST similarity searches with the human OGC amino acid sequence against the *C. elegans* proteome showed the second best match as the uncharacterized protein K11G12.5, predicted to be a malate carrier. In this case, the expect value was 8e^−50^, amino acid identity was 40% and similarities were 57%. Identical amino acids are shown in black, similarities are shown in grey.
(TIF)

Figure S2 **Starvation increases MISC-1 expression.** We observed an increase in MISC-1::GFP reporter expression in animals that were placed for 24 h on plates that contained no food, compared to animals that were fed *ad libitum* on plates streaked with *E. coli* OP50. The increase in reporter expression upon starvation was especially noticeable in the posterior bulb of the pharynx (outlined in upper right panel) and in the posterior intestine (outlined in lower right panel).
(TIF)

Figure S3 **Evaluation of** ***misc-1*** **and** ***OGC*** **knock-down.** (A) Synchronized L1 larvae were treated with either empty vector control (L4440) or *misc-1* RNAi vector until day 1 of adulthood. We performed a Western blot on the proteins extracted from these strains with our MISC-1 antibody. The results show a ∼84% down-regulation of MISC-1 levels upon *misc-1* RNAi treatment. (B) Quantitative Real-Time PCR (qRT-PCR) was performed to assess the extent of *OGC* knock-down in HEK293 cells treated with *OGC* siRNA. As a control, we transfected cells with a mammalian X-Scramble siRNA vector. *OGC* expression levels were normalized to *Actβ*. Our results indicate that *OGC* siRNA reduced target gene expression by ∼80%. Error bars: ±S.D.
(TIF)

Figure S4 ***misc-1*** **knock-down does not affect muscle morphology.** Control RNAi and *misc-1* RNAi on a transgenic strain carrying a *myo-3*p::*myo-3*::*gfp*. This transgene is expressed in muscles and provides a tool to assess muscle morphology. These confocal images show that muscle morphology in *misc-1* RNAi-treated worms is indistinguishable from that of control RNAi-treated worms. The mitochondrial fragmentation phenotype observed in Figure 2B is therefore caused by a specific effect of *misc-1* RNAi on mitochondria, not on muscle structure.
(TIF)

Figure S5 ***misc-1*** **RNAi does not compromise the ability of the somatic gonad to remove apoptotic corpses.** Control RNAi and *misc-1* RNAi treatment on a transgenic strain carrying CED-1::GFP are shown. This fluorescent reporter allows visualization of the apoptotic corpses being engulfed and removed by the somatic sheath cells (arrows). We observed a two-fold increase in the number of engulfing events per gonad arm in worms treated with *misc-1* RNAi, compared to control. This result suggests that the increase in apoptotic events observed in Fig. 4A–B was due to an effect of *misc-1* on apoptosis, and was not caused by a defect in the mechanism of cell corpse removal.
(TIF)

Figure S6 **MISC-1 interacts with apoptotic proteins in** ***C. elegans*** **.** Co-immunoprecipitation experiments performed in wild type N2 (upper panel) and in a transgenic line expressing MISC-1::GFP (lower panel). MISC-1 was shown to interact with CED-9 and ANT. The latter is an integral component of the MPTP. Our data suggest that MISC-1 is a novel component of the MPTP and therefore an important player in the induction of apoptosis. Antibodies used: α-CED-9: sc-33737, Santa Cruz Biotechnology, Santa Cruz CA, USA; α-GFP: ab6556, Abcam Inc., Cambridge MA, USA. See Methods section for other antibodies.
(TIF)
