# Supplementary figures and images for: MISC-1/OGC Links Mitochondrial Metabolism, Apoptosis and Insulin Secretion
Source: PLoS One. 2011 Mar 23;6(3):e17827. doi: 10.1371/journal.pone.0017827 (PMC3063170; doi:10.1371/journal.pone.0017827)

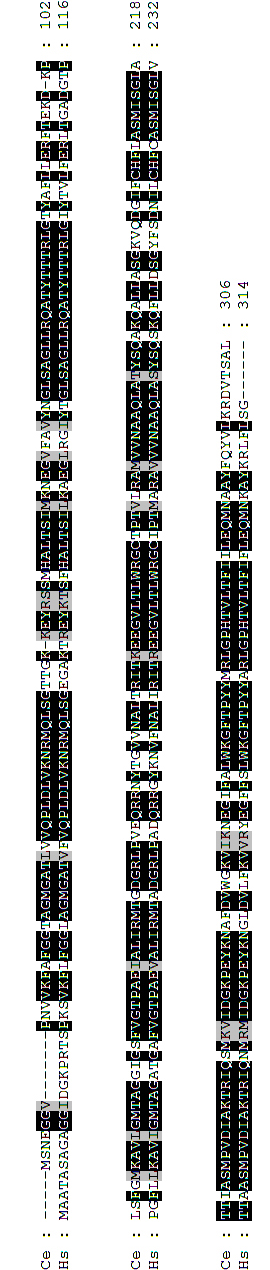

Supplement: Figure S1 — Amino acid conservation between C. elegans MISC-1 and human OGC. MISC-1 and OGC share 72% identity and 80% similarity (e-value = 8e−105) at the amino acid level. The two proteins are best reciprocal matches. When doing BLAST similarity searches with the MISC-1 sequence, the second best human match is the dicarboxylate carrier, with an expect value of 2e−51, amino acid identity of 39% and similarity of 57%. BLAST similarity searches with the human OGC amino acid sequence against the C. elegans proteome showed the second best match as the uncharacterized protein K11G12.5, predicted to be a malate carrier. In this case, the expect value was 8e−50, amino acid identity was 40% and similarities were 57%. Identical amino acids are shown in black, similarities are shown in grey. (TIF) [file pone.0017827.s001.tif]

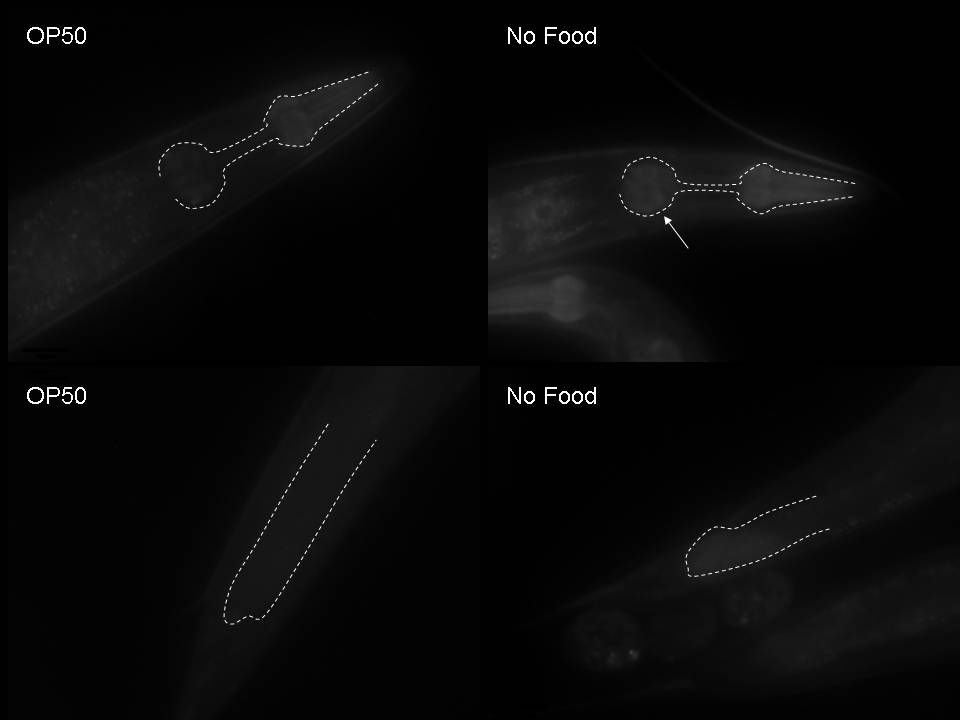

Supplement: Figure S2 — Starvation increases MISC-1 expression. We observed an increase in MISC-1::GFP reporter expression in animals that were placed for 24 h on plates that contained no food, compared to animals that were fed ad libitum on plates streaked with E. coli OP50. The increase in reporter expression upon starvation was especially noticeable in the posterior bulb of the pharynx (outlined in upper right panel) and in the posterior intestine (outlined in lower right panel). (TIF) [file pone.0017827.s002.tif]

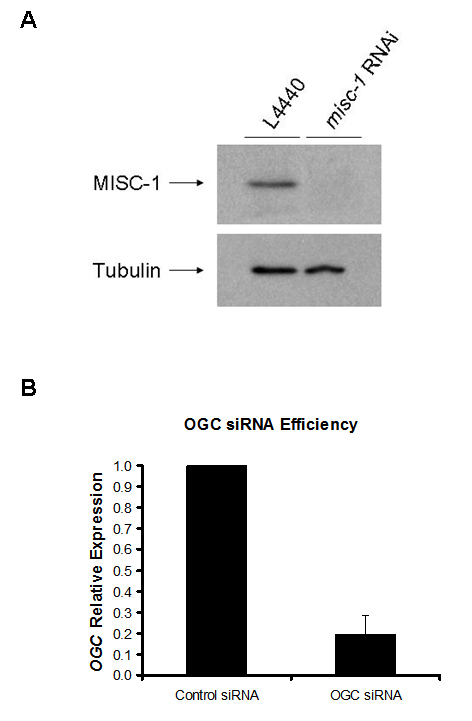

Supplement: Figure S3 — Evaluation of misc-1 and OGC knock-down. (A) Synchronized L1 larvae were treated with either empty vector control (L4440) or misc-1 RNAi vector until day 1 of adulthood. We performed a Western blot on the proteins extracted from these strains with our MISC-1 antibody. The results show a ∼84% down-regulation of MISC-1 levels upon misc-1 RNAi treatment. (B) Quantitative Real-Time PCR (qRT-PCR) was performed to assess the extent of OGC knock-down in HEK293 cells treated with OGC siRNA. As a control, we transfected cells with a mammalian X-Scramble siRNA vector. OGC expression levels were normalized to Actβ. Our results indicate that OGC siRNA reduced target gene expression by ∼80%. Error bars: ±S.D. (TIF) [file pone.0017827.s003.tif]

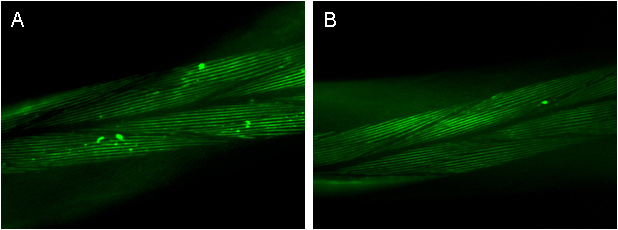

Supplement: Figure S4 — misc-1 knock-down does not affect muscle morphology. Control RNAi and misc-1 RNAi on a transgenic strain carrying a myo-3p::myo-3::gfp. This transgene is expressed in muscles and provides a tool to assess muscle morphology. These confocal images show that muscle morphology in misc-1 RNAi-treated worms is indistinguishable from that of control RNAi-treated worms. The mitochondrial fragmentation phenotype observed in Figure 2B is therefore caused by a specific effect of misc-1 RNAi on mitochondria, not on muscle structure. (TIF) [file pone.0017827.s004.tif]

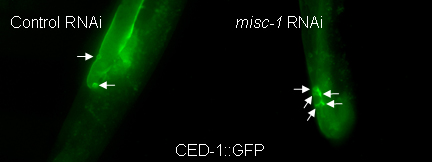

Supplement: Figure S5 — misc-1 RNAi does not compromise the ability of the somatic gonad to remove apoptotic corpses. Control RNAi and misc-1 RNAi treatment on a transgenic strain carrying CED-1::GFP are shown. This fluorescent reporter allows visualization of the apoptotic corpses being engulfed and removed by the somatic sheath cells (arrows). We observed a two-fold increase in the number of engulfing events per gonad arm in worms treated with misc-1 RNAi, compared to control. This result suggests that the increase in apoptotic events observed in Fig. 4A–B was due to an effect of misc-1 on apoptosis, and was not caused by a defect in the mechanism of cell corpse removal. (TIF) [file pone.0017827.s005.tif]

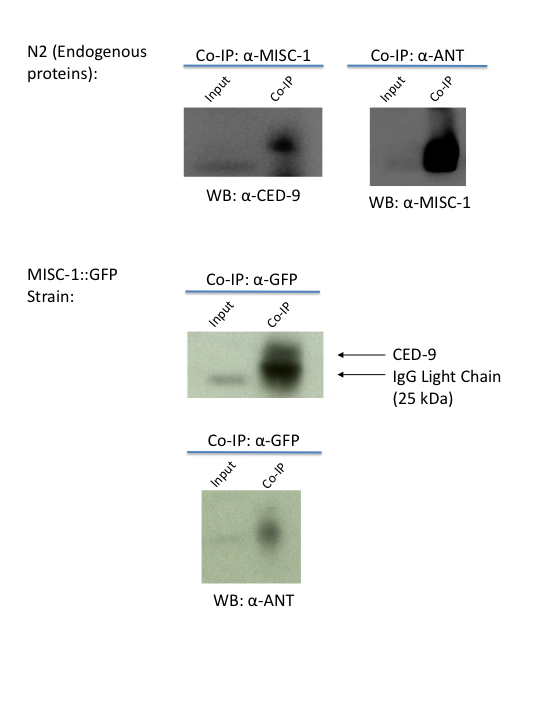

Supplement: Figure S6 — MISC-1 interacts with apoptotic proteins in C. elegans . Co-immunoprecipitation experiments performed in wild type N2 (upper panel) and in a transgenic line expressing MISC-1::GFP (lower panel). MISC-1 was shown to interact with CED-9 and ANT. The latter is an integral component of the MPTP. Our data suggest that MISC-1 is a novel component of the MPTP and therefore an important player in the induction of apoptosis. Antibodies used: α-CED-9: sc-33737, Santa Cruz Biotechnology, Santa Cruz CA, USA; α-GFP: ab6556, Abcam Inc., Cambridge MA, USA. See Methods section for other antibodies. (TIF) [file pone.0017827.s006.tif]
